# Supplementary material for: Exploring person-specific associations of situational motivation and readiness with leisure-time physical activity effort and experience
Source: PLoS One. 2024 Jul 18;19(7):e0307369. doi: 10.1371/journal.pone.0307369 (PMC11257293; doi:10.1371/journal.pone.0307369)
Supplement: Attachment — Submitted filename: review_PLosOne_2024.pdf [file pone.0307369.s003.pdf]

Manuscript ID: PONE-D-24-08085

The manuscript titled "Perceptual responses to high- and moderate-intensity interval exercise in adolescents" Exploring heterogeneity in person-specific associations of situational motivation and readiness with leisure-time physical activity duration, intensity, and affective valence'. Overall, the manuscript is very well-written and clearly describes the reason for the research, what gap the research is filling, and accurately concludes with the impact of the results on the field. The research question and results would be of interest to the readers of the journal. My enthusiasm for the paper remains despite the following issues that need to be addressed as highlighted below:

Is there any specific reason why the researcher chose 10 weeks for conducting the observation? Since the participants who enrolled in this study underwent multiple procedures (e.g. answering the questionnaire pre-post activity) across the 10 weeks, this situation can lead to an enforcement type of situation rather than a volitional-based PA situation. Consequently, most probably the participants come out with any kind of activity just to complete the task. How did the researcher control this possible situation? If this happens, the intended volitional LTPA effort and affective experiences will be compromised. Any comments?

Line 138: why a minimum of 60 minutes per week of MVPA was chosen as one of the intrinsic criteria of the participants? Any specific reason?

Line 144-148: it would be useful to include the sample size calculation to provide the readers with the minimum number of participants required for this study. What indicator that the researcher uses to decide whether the number of participants was sufficient for the analysis based on the snowball sampling technique?

Line 227 – 232: Evidence has shown that post-affective responses/experience did not reflect on individual exercise/PA adherence and no such thing as recalled in-task affective responses. Affective responses measured via a single-item scale FS usually conducted during the exercise/PA, not after the completion of the exercise/PA. This is because post-exercise feelings will lead to homogeneous pleasurable feelings regardless of the type and intensity of the activity. Any comments?
