# Supplementary material for: Exploring person-specific associations of situational motivation and readiness with leisure-time physical activity effort and experience
Source: PLoS One. 2024 Jul 18;19(7):e0307369. doi: 10.1371/journal.pone.0307369 (PMC11257293; doi:10.1371/journal.pone.0307369)
Supplement: Attachment — Submitted filename: Response to Reviewer Comments.docx [file pone.0307369.s004.docx]

We would like to sincerely thank the reviewers for their insights and critiques. We have prepared point-by-point responses (nonbolded) to each comment (bolded) in this document. The location of changes in the manuscript are identified here by page and line number. All revisions in the manuscript are highlighted using track changes. We believe that we have thoroughly addressed all concerns and have consequently improved the strength and clarity of our manuscript.

Note: Due to continuity errors in line numbering when using track changes, all page/line numbers referred to below are specific to the ‘clean copy’ of the revised manuscript

**Reviewer #1**

**I would like to thank for the opportunity to review this highly interesting manuscript. Overall, this manuscript is well written, and it presents important topic of motivation and physical activity. I have only some comments to further improve the quality of this manuscript.** We appreciate the reviewer’s interest and support for this manuscript.

**Title: Overall, the title of this manuscript is an accurate presentation of this manuscript content. However, perhaps Authors could shorten their title in terms of clarity.** We have incorporated a clearer and shorter title (122 characters/15 words vs. 156 characters/19 words). The revised title reads as follows:

“Exploring person-specific associations of situational motivation and readiness with leisure-time physical activity effort and experience” *(Page 1; Lines 1-2)*

**Abstract: The abstract of this manuscript provides the most important information for the reader. However, one issue of this manuscript is that in some cases Authors use very long sentences that might be hard to follow for the reader. For example, the last sentence of the abstract is written on five lines.** We have reviewed the abstract and, where possible, reduced longer sentence structures and used clearer wording to improve readability and impact. The overall word and character counts have been reduced by approximately 15% each and remain within the journal guidelines for length.

**Main text: Although the introduction of this manuscript is well written, some concepts are not introduced, and some findings are presented in too much detail. Authors are recommended to provide more general introduction of previous findings and avoid too many details about specific previous research papers. Authors are using scales that measure different forms of motivation from the self-determination theory but in the introduction, there is no information at all about the self-determination theory.** Details regarding study methods and/or results have been removed regarding references 10 and 11 *(Page 4; Lines 58-59)*, 22 and 23 *(Page 4; Lines 82-85)*, and 28 *(Page 5; Lines 94-100)*. We also now reference Self-Determination Theory to provide examples of physical activity determinants in a more concise manner and to better support later methods *(Page 3, Lines 59-61)*.

**Methods section is a bit messy. For example, there is information under the study design that should be under the data analysis section. Participants are poorly described and there are several paragraphs about participants (could be only one).** We have minimized the detail in the study design section to improve readability. As not all enrolled participants provided sufficient data to be retained in the analysis, a more thorough description of participants was provided in the first paragraph of the results. We felt that by providing a full description of the sample before describing the procedures used to determine inclusion in the final analyses would confuse the readers. To reduce the potential for confusion and length of the section, we have also combined and made more concise the recruitment, enrollment, and orientation procedures to minimize the number of paragraphs dedicated to participant-related activities.

**Overall, the methods could be organized better.**

In addition to the changes listed in response to the above comment, we have also made the following edits to improve organization and readability:

1. re-allocated information from the ‘approaches for participant retention’ section and deleted the subheading.
2. Minimized detail and combined the first two paragraphs under ‘statistical and visual analysis’.
3. Reduced instances where a statement may be perceived as overly wordy.

**Overall, the results and described in a great detail.** We feel that the level of detail is sufficient to communicate a relatively complex view of how data are interpreted based on differing levels of analyses.

**Tables are formatted in different style.** We have added gray shading to Table 1 to align with formatting in Table 2.

**The quality of figures could be improved.** We have reviewed all of the images, which show a good resolution in the PDF form, which were included in the original submission. Some pixelation only occurs when zooming in at 400%, but not other problems were detected. If there remain any quality concerns, we would be very grateful for specific information from the reviewer or editor to guide the necessary improvements.

**Overall, the discussion of this manuscript is well written. However, the discussion could be structured better.** To improve the structure of the discussion, we have added subheadings to guide the reader more clearly in the subsequent content (e.g., ‘summary of findings’, ‘variability in reported LTPA characteristics’, ‘level of analysis and differing data interpretation’, ‘future directions in data analysis and intervention development’, ‘limitations’, and ‘conclusions’). Where reasonable, we have also split longer paragraphs into two for improved readability and understanding.

**Also, more suggestions for future research could be provided. Specific recommendations for future motivational interventions could be given with the aim to increase physical activity behavior. There are several recent high level classification systems of motivational behaviors recommended in self-determination theory interventions that could be used to design need-supportive motivational intervention with the aim to increase physical activity behavior.** We have added a new discussion paragraph, under ‘future directions in data analysis and intervention development’. This paragraph is more specific to intervention-related research and incorporates thoughts on motivation and behavior-change techniques with the following statements:

“Given that enactment of FNLP – the exercise programming model that informed study variables – is hypothesized to foster more self-determined forms of motivation [28], intervention development efforts should incorporate terms and definitions according to the taxonomy of motivation and behavior-change techniques proposed by Teixeira and colleagues (2020) for health promotion contexts [71]. For example, FNLP ‘provides choice’ and encourages self-experimentation [28] as *autonomy-support techniques* to ‘assist in setting optimal challenge’ based on situational motivation and readiness states as a *competence-support technique*.” *(Page 24 and 25, Lines 504-519)*

**Reviewer #2**

**The manuscript titled " Exploring heterogeneity in person-specific associations of situational motivation and readiness with leisure-time physical activity duration, intensity, and affective valence’. Overall, the manuscript is very well-written and clearly describes the reason for the research, what gap the research is filling, and accurately concludes with the impact of the results on the field. The research question and results would be of interest to the readers of the journal. My enthusiasm for the paper remains despite the following issues that need to be addressed as highlighted below.** We appreciate the reviewer’s enthusiasm for the work.

**Is there any specific reason why the researcher chose 10 weeks for conducting the observation?**

We elected to capture data across 10 weeks so that we could describe physical activity behaviors and experiences that people engage in across more natural ebbs and flows of life, which may be missed within a more traditional EMA timeframe (i.e., 14 days). For example, behavior during a vacation is likely to differ from behavior performed during periods of higher demand (e.g., mid-term exams) in terms of frequency, effort, and experience. In other words, too short of a time-frame may result in an inaccurate interpretation that variables are relatively stable and uniform.

Also, aligned with our rationale for the 60-min of MVPA inclusion criteria and sample size considerations (see responses to the next two comments), we assumed individuals would report a minimum of 1 to 2 LTPA sessions per week to ensure a substantial minimum of around 10 to 15 events per person over the measurement period. This is comparable to the mean number of responses in other EMA study in physical activity research and affective response to exercise applying intensive longitudinal assessment over a few days with multi daily assessment (see for example the review by Liao et al., 2016)

We also considered our study design from a practical viewpoint. In Germany, the typical length of exercise courses in preventive settings, which are reimbursed by health insurance companies, is 8 to 10 weeks with 1 or 2 sessions per week. Those preventive exercise settings can be an important field for implementing person-adaptive exercise approaches (i.e., flexible nonlinear periodization), for which this study provides initial empirical insights for main tenets of this conceptual approach.

**Since the participants who enrolled in this study underwent multiple procedures (e.g. answering the questionnaire pre-post activity) across the 10 weeks, this situation can lead to an enforcement type of situation rather than a volitional-based PA situation. Consequently, most probably the participants come out with any kind of activity just to complete the task. How did the researcher control this possible situation? If this happens, the intended volitional LTPA effort and affective experiences will be compromised. Any comments?**

As indicated in the previous response, the focus of this study was on volitional LTPA and its possible dynamics with respect to event-to-event variations over the course of multi-week period. As the weekly frequency of LTPA is lower compared to other types of PA (e.g. everyday PA in various non-leisure time domains) or other health behaviors with daily occurrence, by the ten-week assessment period we wanted to ensure that the participants can keep their usual (‘natural) LTPA behavior as best as possible and do not feel pressure to be more active than they usual would be while still allowing us to capture a usable amount of data. Thus, the instruction for the participants was accordingly and highlighted that participants should behave as usual during the study period. The participants were not given a target or minimum number of sessions to record and participants were not provided any incentive to engage in the research procedures.

However, we cannot ultimately rule out that the study procedures produces reactivity processes of the participants (e.g. doing more LTPA sessions as usual) and that this also partially hinder the volitional nature of the PA behavior under study. Thus, we have added the following statements to the methods (reflecting language already in the abstract) and limitations sections:

“Participants were instructed to continue their usual pattern of physical activity behavior and initiate event-contingent surveys…” *(Page 7; Lines 143-146)*

“No incentives or compensation were provided for engaging in research activities. ” *(Page 7; Lines 148-149)*

“While participants in the current sample were explicitly asked to maintain normal patterns of behavior and were not given a minimum number of sessions to perform or incentivized to initiate EMA reports, we cannot with certainty rule out the possibility for reactivity. It is important to acknowledge that perceived enforcement of behavior (e.g., performing additional or undesired LTPA due to being observed by researchers) would compromise the aim to understand volitional behavior and experiential aspects thereof. In future studies, it may be beneficial to conduct debriefing assessments (quantitative surveys or qualitative interviews) to understand the potential for reactivity and determine strategies to minimize such effects.“ *(Page 26; Lines 544-552)*

**Line 138: why a minimum of 60 minutes per week of MVPA was chosen as one of the intrinsic criteria of the participants? Any specific reason?**

Our research group had previously conducted a signal-contingent EMA study (<https://trace.tennessee.edu/pursuit/vol9/iss1/1/>), such that individuals were prompted four times per day, across 14 days, to indicate whether or not exercise was performed in the four hours prior to each prompt. For this study, we had no inclusion criterion related to current activity level and found that of the 28 individuals who completed the study, 25% reported between 0 and 2 exercise sessions per week. As the current, event-contingent EMA study was designed to explore pre-activity states and LTPA-related effort and experience, we added the 60-min inclusion criterion to try to ensure that all enrolled participants would report more than zero LTPA sessions per week, in alignment with added sample size considerations detailed in the next response.

**Line 144-148: it would be useful to include the sample size calculation to provide the readers with the minimum number of participants required for this study. What indicator that the researcher uses to decide whether the number of participants was sufficient for the analysis based on the snowball sampling technique?**

Because of the exploratory nature of our study (i.e., p-values are not reported) and the intended person-specific analyses, we did not perform *a priori* sample size calculations, which are directed on identifying the optimal sample sizes for confirmatory tests of statistical significance.

We primarily made considerations regarding the required minimum number of LTPA events per person in order to capture potential event-to-event variations, if they actually occur. For example, if, based on person-specific assessments, an identifiable subgroup emerged (e.g., individuals for whom stronger motivations for stress relief were related to lower ratings of perceived exertion), person-centered analyses (e.g. repeated measures correlation) could then be conducted to provide usable estimates of effects. In this regard, to provide insight into sample size considerations, we have added the following statement:

“Though confirmatory statistics were not conducted for this study, recruitment goals and enrollment duration were guided by repeated measures correlation power curves [32], such that a minimum of 10 individuals, each with 10 within-person data points (i.e., 1 report of LTPA per week) would be required to detect at least medium (r=0.3) effects with 80% power.” *(Page 6-7; Lines 136-139)*

We anticipate that the work here will provide more information to guide future power analyses in subsequent research.

**Line 227 – 232: Evidence has shown that post-affective responses/experience did not reflect on individual exercise/PA adherence and no such thing as recalled in-task affective responses. Affective responses measured via a single-item scale FS usually conducted during the exercise/PA, not after the completion of the exercise/PA. This is because post-exercise feelings will lead to homogeneous pleasurable feelings regardless of the type and intensity of the activity. Any comments?**

The reviewer is correct, asking individuals to provide momentary ratings of affect (i.e., “how are you feeling right now?”) in a post-activity state is likely to result in relatively uniform, positive ratings due to the removal of a potentially aversive stimulus (e.g., exercise). With this awareness and study limitations (i.e., we were not able to pre-program EMAs to be distributed once a pre-activity report was completed), we elected to use a more specific prompt so that individuals were asked to recall specifically how they felt while under exertion. We have added additional methodological detail to clarify this approach:

“Rather than using ‘right now’ framing (which is likely to capture a rebound effect [50]), instructions were provided to elicit ratings as representative as possible of affective experiences while under exertion (‘*Overall, how pleasant or unpleasant did you feel WHILE you were physically active*’), as true in-task measures were not able to be captured in this study. This approach had been used previously within recreation center exit surveys [61].” *(Page 9; Lines: 194-198)*

In acknowledgment that recalling in-task feelings is not a complete substitute for measuring true in-task responses have made two additional revisions: we (1) removed references to ‘in-task’ and now refer to the construct as ‘recalled affective valence’; (2) added additional statements to the limitations section:

“A second limitation is that results based on event-contingent reporting of volitional LTPA may be skewed more positive, as individuals could be less likely to initiate reports in response to strongly negative experiences. Additionally, despite the prompts specifically asking individuals to recall feelings during activity, capturing data in the post-activity stay may still yield higher-than-actual ratings of pleasure. While this approach had been used previously [61] to overcome limitations in collecting in-task data, it will be necessary to replicate these methods in intervention or treatment settings, paired with wearable devices or direct observation to more accurately monitor physical stimuli and implement approaches to capture actual in-task ratings of affect.” *(Page 25; Lines 527-535)*
