# Supplementary material for: Exploring person-specific associations of situational motivation and readiness with leisure-time physical activity effort and experience
Source: PLoS One. 2024 Jul 18;19(7):e0307369. doi: 10.1371/journal.pone.0307369 (PMC11257293; doi:10.1371/journal.pone.0307369)
Supplement: Attachment — Submitted filename: Response to Reviewer Comments.docx [file pone.0307369.s005.docx]

We would like to sincerely thank the reviewers for assessing our revised manuscript. We have prepared point-by-point responses (nonbolded) to each comment (bolded) in this document. The location of changes in the manuscript are identified here by page and line number. All revisions in the manuscript are highlighted using track changes. We believe that we have thoroughly addressed the remaining point by Reviewer #1, which further improves our manuscript regarding use of relevant and contemporary literature.

Note: Due to continuity errors in line numbering when using track changes, all page/line numbers referred to below are specific to the ‘clean copy’ of the revised manuscript.

**Reviewer #1**

**Reviewer #1: Overall, Authors have done well job on revising their manuscript. I have only one comment. Specifically, Authors have argued that intervention development efforts should incorporate terms and definitions according to the taxonomy of motivation and behavior-change techniques proposed by Teixeira and colleagues (2020) for health promotion contexts. I would like to add that for educational context, a classification system of motivational behaviors by Ahmadi and colleagues (2023) was recently developed. Health-related messages are often provided to children in the educational context (e.g., during physical education classes). The advantage of Ahmadi and colleagues (2023) classification system of motivational behaviors is that it also lists need-thwarting behaviors. It is important to not only provide need support, but also to avoid need thwarting, because need satisfaction and need thwarting are related to adaptive and maladaptive outcomes via separate pathways (Haerens et al., 2015). Thus, it is important to adopt need-supportive behaviors and at the same time avoid need-thwarting behaviors. There are also more advantages of Ahmadi and colleagues (2023) classification system of motivational behaviors such as more experts were involved in Ahmadi and colleagues (2023) Delphi panel compared to Teixeira and colleagues (2020) study (34 vs 18 experts).

Ahmadi, A., Noetel, M., Parker, P., Ryan, R. M., Ntoumanis, N., Reeve, J., Beauchamp, M., Dicke, T., Yeung, A., Ahmadi, M., Bartholomew, K., Chiu, T. K. F., Curran, T., Erturan, G., Flunger, B., Frederick, C., Froiland, J. M., González-Cutre, D., Haerens, L., . . . Lonsdale, C. (2023). A classification system for teachers’ motivational behaviors recommended in self-determination theory interventions. Journal of Educational Psychology, 115(8), 1158–1176.** [**https://doi.org/10.1037/edu0000783**](https://doi.org/10.1037/edu0000783) **Haerens, L., Aelterman, N., Vansteenkiste, M., Soenens, B., & Van Petegem, S. (2015). Do perceived autonomy-supportive and controlling teaching relate to physical education students’ motivational experiences through unique pathways? Distinguishing between the bright and dark side of motivation. Psychology of Sport and Exercise, 16, 26–36.** [**https://doi.org/10.1016/j.psychsport.2014.08.013**](https://doi.org/10.1016/j.psychsport.2014.08.013)

We strongly appreciate the reviewer for alerting us to the taxonomy developed by Ahmadi and colleagues. This taxonomy is not only informative, but complementary considering that our proposed programming strategy (flexible nonlinear periodization; FNLP) is likely to be delivered by professionals who also strive to educate exercisers under their guidance. We have added the following statements (with both provided references) in the revised manuscript:

“As FNLP may be implemented by a professional (e.g., certified exercise physiologist, behavioral interventionist) who educates patients or clients, a complementary taxonomy proposed by Ahmadi and colleagues (2023) will also benefit intervention development and assessment efforts [72]. This taxonomy importantly specifies behaviors *of the educator* that can support or thwart autonomous motivation, in recognition outcomes are differently impacted via separate pathways (e.g., psychological need satisfaction and need frustration) [73].” *(Page 25; Lines 518-523)*

**Reviewer #3**

**Overall
This study investigates the association between psychological constructs (e.g., situational motivation and readiness) and leisure-time physical activity (PA). Specifically, that authors explore how idiographic analytical approaches might be used when EMA data is available to assess individualized associations between psychological constructs and PA. The authors note differences between idiographic and nomothetic approaches and discuss how idiographic approaches may be useful for patient engagement and tailoring of interventions. This work is a good case study for behavioral interventionists interested in utilizing technology and tailoring interventions on the potential use of idiographic methods.
The authors responded sufficiently to previous reviewer comments and appropriately discuss the limitations of this work and its implications for future research.
Abstract
The abstract is clear.
Introduction
The motivation for the work is described in a logical sequence and the author’s framing of the work as a proof of concept appears acceptable.
Methods
The instruction given to participants are clearly described. The sample size justification is appropriate for a study of this type. All measures are discussed with sufficient references.
Results
The results are sufficiently explained.
Discussion
References provided in the discussion are helpful to readers who are interested in further applying these idiographic methods.
Tables/Figure
Figures are of publishable quality. Legends are provided as appropriate.**

We thank the reviewer for assessing our manuscript relative to the prior revision. We have made no additional changes to the abstract, introduction, methods, results, or figures.
